# Tissue Doppler imaging for diagnosis of coronary artery disease: a systematic review and meta-analysis

**DOI:** 10.1186/1476-7120-10-47

**Published:** 2012-11-30

**Authors:** Rajender Agarwal, Priyanka Gosain, James N Kirkpatrick, Tareq Alyousef, Rami Doukky, Gurpreet Singh, Craig A Umscheid

**Affiliations:** 1 Department of Medicine, John H. Stroger Jr. Hospital of Cook County, Chicago, IL, USA; 2 Cardiovascular Division, Department of Medicine, University of Pennsylvania Perelman School of Medicine, Philadelphia, PA, USA; 3 Division of Cardiology, John H. Stroger Jr. Hospital of Cook County, Chicago, IL, USA; 4 Section of Cardiology, Rush University Medical Center, Chicago, IL, USA; 5 Division of General Internal Medicine, Department of Medicine, University of Pennsylvania Perelman School of Medicine, Philadelphia, PA, USA; 6 Center for Clinical Epidemiology and Biostatistics, University of Pennsylvania Perelman School of Medicine, Philadelphia, PA, USA; 7 Leonard Davis Institute of Health Economics, University of Pennsylvania, Philadelphia, PA, USA; 8 Center for Evidence-based Practice, University of Pennsylvania Health System, Philadelphia, PA, USA

**Keywords:** Systematic review, Meta-analysis, Tissue Doppler, Echocardiography, Coronary artery disease

## Abstract

Global and regional left ventricular (LV) systolic dysfunction is a marker of coronary artery disease (CAD), which is conventionally assessed using two-dimensional echocardiography. Tissue Doppler imaging (TDI) has emerged as an adjunct tool in the diagnosis of regional wall motion abnormalities from CAD. We performed a systematic review and meta-analysis to assess the efficacy of TDI indices in the diagnosis of CAD. We searched MEDLINE and the Cochrane Library for controlled studies comparing TDI measurements in those with and without CAD as confirmed by coronary angiography. Meta-analyses of mean differences in TDI velocities between these populations were performed. Screening of titles and abstracts followed by full-text screening identified 8 studies. At rest, TDI was associated with a significant decrease in the pooled maximum systolic velocity among CAD patients compared to those without CAD [mean difference (MD): -0.66; 95% confidence interval (CI): -0.98 to −0.34]. There were no significant differences in maximum early and late diastolic velocities. Post-stress, TDI was associated with a significant decrease in maximum early diastolic velocity (MD: -1.91; 95% CI: -2.74 to −1.09) and maximum late diastolic velocity (MD: -1.57; 95% CI: -2.95 to −0.18) among CAD patients compared to those without CAD. There was no significant difference in maximum systolic velocity post-stress. Our results suggest that TDI may have a role in the evaluation of CAD. Future studies should evaluate the incremental value of TDI velocities over LV ejection fraction and two dimensional wall motion analysis in the detection of CAD and assessment of its severity. (Word Count: 249)

## Introduction

Coronary artery disease (CAD) is responsible for one out of every six deaths in the United States. Each year, approximately 800,000 Americans have a new coronary event resulting in approximately one death per minute [1]. This extraordinary burden of disease necessitates early diagnosis and treatment.

Non-invasive imaging tests like radionuclide imaging and stress echocardiography are frequently used in clinical practice for detection and evaluation of CAD [2]. Global and regional left ventricular (LV) systolic function is an important marker of CAD in stress echocardiography, which is conventionally assessed using two-dimensional echocardiography. In 1989, the technique of tissue Doppler imaging (TDI) emerged as a potential modality for assessing systolic and diastolic LV performance [3-5]. TDI visualizes myocardial velocities by measuring low-frequency, high-amplitude signals of myocardial motion. TDI is done using either the spectral pulsed-wave or the color-coded pulsed-wave technique. Spectral Doppler measures the instantaneous velocity in a sample volume of the region of interest, while color-coded Doppler allows simultaneous sampling of the entire ultrasound sector [6-8].

Most of the studies of TDI have employed the technique for measuring LV diastolic function. Other investigators have applied TDI to measure resting or post-stress velocities of various myocardial segments of the LV as an adjunct tool in the diagnosis of regional wall motion abnormalities from CAD. However, there has not been a consensus on the value of these techniques in the diagnosis of CAD. In this paper, we perform a systematic review and meta-analysis to assess the efficacy of TDI indices in the diagnosis of CAD.

## Methods

### Study selection

We searched MEDLINE (inception to June 2012) and the Cochrane Library (inception to June 2012) using keywords and/or medical subject headings (MeSH) for TDI and CAD. The detailed search strategy for MEDLINE is presented in Table 1. Titles and abstracts of the references identified were screened by a single reviewer. This was followed by full-text review by two independent reviewers of those articles meeting inclusion criteria. Disagreements were resolved by consensus. We included in our analysis all controlled trials that: 1) were published in English, 2) compared TDI measurements (spectral pulsed wave or color-coded pulsed wave) in those with and without CAD as confirmed by coronary angiography, and 3) evaluated at least one of the following measurements - maximum systolic velocity, maximum early diastolic velocity or maximum late diastolic velocity.

**Table 1:** Search strategy for including studies

***PHASE 1: SEARCH TERMS FOR TISSUE DOPPLER***	
1	tissue adj doppler
2	tissue adj velocity adj imaging
3	(TDI or TVI).mp.
4	or/1-3
***PHASE 2: SEARCH TERMS FOR CORONARY ARTERY DISEASE***	
5	exp Coronary Artery Disease/
6	exp Atherosclerosis/
7	(coronary adj artery adj disease).mp.
8	CAD.mp.
9	or/5-8
***PHASE 3: COMBINING THE SEARCHES AND RESTRICTING TO ENGLISH***	
10	4 and 9
11	limit 10 to english language

### Data extraction

We extracted data on study design, intervention, patient population, size of the study sample, and our outcome measures. The primary outcomes of interest were the mean differences in the following variables as measured by TDI in those with and without CAD: maximum systolic velocity, maximum early diastolic velocity, and maximum late diastolic velocity. In addition we also extracted data on mean differences in E/E' (the ratio of early diastolic transmitral flow velocity and early diastolic tissue velocity) and E'/A' (the ratio of early and late diastolic tissue velocity) as measured by TDI in those with and without CAD. For all outcomes, data was extracted as means and standard deviations.

### Statistical analyses

We performed meta-analyses of the included controlled studies using the Mantel-Haenszel procedure that assumes a fixed effect size [9]. We measured heterogeneity between study results using the *I*^*2*^ statistic [10]. We defined significant heterogeneity as an *I*^*2*^ statistic ≥ 50% which would imply real differences between study results that would not be explained by chance alone. In the presence of significant heterogeneity, we performed additional meta-analyses using the random-effects model that produces more conservative estimates of effect [11]. The estimates used in our meta-analyses were mean differences (MD) and 95% confidence intervals (CI) in TDI measurements for our measures of interest in those with and without CAD. Sensitivity analyses were performed using only those studies that verified both the presence and absence of CAD with angiography.

## Results

### Study selection

**Figure 1:**
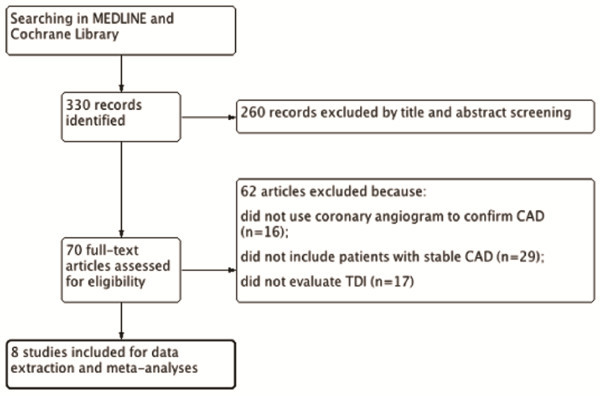
Flow diagram for selection of included studies.

Our literature search initially yielded 330 references. Screening of titles and abstracts followed by full-text screening ultimately identified 8 studies [12-19] that met our pre-defined inclusion criteria (Figure 1).

### Study characteristics

Combined, the 8 studies included 568 patients. Details of the study populations, segments in which TDI velocities were studied and the echocardiography machine used are shown in Table 2. No randomized controlled trials were identified; all studies were prospective controlled studies. CAD patients had significant disease confirmed by angiography in all studies. Patients without significant CAD either had no obstructive CAD on angiography or had negative results on non-invasive stress testing.

**Table 2:** Study characteristics

**Author, Year (Reference)**	**Study design**	**CAD (N)**	**No CAD (N)**	**Segments studied**	**Type of tissue doppler**	**Machine used**
Bolognesi, 2001 [12]	Prospective controlled study	Stable effort angina without previous MI and with normal EF, CAD confirmed with angiography(16)	Negative noninvasive coronary stress tests and normal electrocardiographic and echocardiographic findings in volunteers undergoing cardiac catheterization (6)	Apical four chamber view: septal and anterolateral	Not reported	Toshiba 380, Rome, Italy
				Apical two chamber view: anterior and inferior		
Bruch, 1999 [13]	Prospective controlled study	CAD on angiogram (>70% LAD), no previous MI or cardiac surgery and no echo evidence of regional or global wall motion abnormalities (17)	Normal resting electrocardiogram and a normal regional and global systolic left ventricular function on echocardiogram (20)	Apical four chamber view: midseptal and midlateral	Color-coded pulsed-wave	Toshiba 380, Rome, Italy
Dounis, 2006 [14]	Prospective controlled study	1-2 vessel disease, confirmed by angiogram, normal EF and sinus rhythm (17)	No CAD confirmed by either angiogram or stress testing, normal EF and sinus rhythm(14)	Apical four-, two- and three-chamber views: septal, anteroseptal, anterior, lateral, posterior and inferior	Spectral pulsed-wave	System V, GE Milwaukee, USA
Hoffmann, 2010 [15]	Prospective controlled study	Significant one, two or three vessel disease included. (47)	Suspected angina pectoris and non significant stenosis on angiogram. (35)	Septal, lateral, inferior, anterior, posterior, and anteroseptal	Color-coded pulsed-wave	Vivid 7, GE Healthcare, Horton, Norway
Madler, 2003 [16]	Prospective controlled study	Significant stenosis (>50%) on coronary angiography (90)	Chest pain with no significant stenosis (<50%)on coronary angiography (59)	Apical four and two chamber view: basal septal, basal anterior, basal lateral and basal inferior and mid septal, mid anterior, mid lateral and mid inferior	Color-coded pulsed-wave	System V, GE Vingmed, Horten, Norway
Tsougos, 2008 [17]	Prospective controlled study	Stenosis >70% of at least one coronary artery (72)	No significant stenosis on angiography (42)	Apical four-chamber view: septal and lateral	Spectral pulsed-wave	Vivid 3, GE Vingmed Horten, Norway
Williams, 2005 [18]	Prospective controlled study	Angiography confirmed >50% stenosis in at least one major coronary artery branch; chronic stable symptoms and normal resting global/regional LV function assessed by left ventriculography and echocardiography (16)	Normal coronary angiography; negative, maximum, Bruce protocol exercise testing, and low probability of coronary artery disease (12)	American Society of Echocardiography 16 segment model	Color-coded pulsed-wave	System V, GE Vingmed, Horten, Norway
Zagatina, 2007 [19]	Prospective controlled study	Significant stenosis in the LAD on angiography (88)	Normal angiogram (17)	Long and short-axis parasternal views and the four and two-chamber apical views: basal and mid-septum, basal and midlateral, inferior and anterior	Spectral pulsed-wave	Hewlett Pacard Sonos 2000

Abbreviations: *CAD* coronary artery disease, *EF* ejection fraction, *LAD* left anterior descending, *LV* left ventricle, *MI* myocardial infarction.

TDI parameters were assessed at baseline in all the studies except one [19] and were assessed after stress testing in 5 studies [14,16-19]. Among the latter, exercise stress testing was performed in 3 studies ( [17-19], dipyridamole stress testing was performed in one study [14] and dobutamine stress testing was performed in one study [16]. Three studies reported data on E/E' [12,13,17] and 4 studies on E'/A' [12-14,17].

### Primary outcomes (Pre-stress)

**Figure 2:**
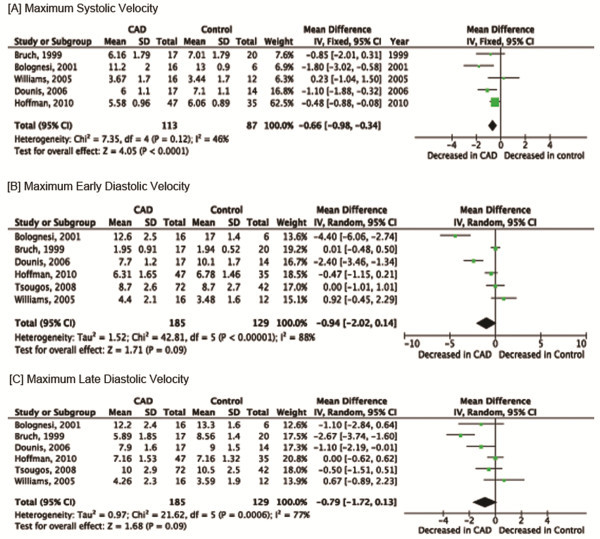
Meta-analysis: Primary outcomes (pre-stress).

TDI was associated with a significant decrease in maximum systolic velocity among CAD patients when compared to those without CAD (MD: -0.66; 95% CI: -0.98 to −0.34). There was no significant heterogeneity among studies (*I*^*2*^ =46%) (Figure 2A). Maximum early diastolic velocity was significantly decreased among CAD patients when compared to those without CAD (MD: -0.46; 95% CI: -0.79 to −0.13). However, *I*^*2*^ was significant at 88%. When random-effects meta-analysis was done, the mean difference between those with and without CAD was not significant (MD: -0.94; 95% CI: -2.02 to 0.14) (Figure 2B). TDI was associated with a significant decrease in maximum late diastolic velocity among CAD patients when compared to those without CAD (MD: -0.63; 95% CI: -1.04 to −0.23; *I*^*2*^ =77%). Because of the significant heterogeneity, a random-effects meta-analysis was performed which did not demonstrate a significant difference (MD: -0.79; 95% CI: -1.72 to 0.13) (Figure 2C).

### Primary outcomes (post-stress)

**Figure 3:**
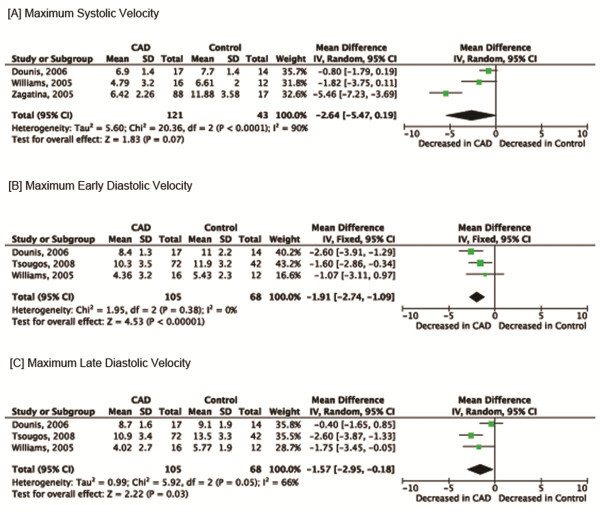
Meta-analysis: Primary outcomes (post-stress).

Meta-analysis demonstrated a significant decrease in maximum systolic velocity among CAD patients when compared to those without CAD (MD: -1.90; 95% CI: -2.69 to −1.11). There was significant heterogeneity (*I*^*2*^ =90%). Differences between patients with and without CAD were not statistically significant with a random-effects meta-analysis (MD: -2.64; 95% CI: -5.47 to 0.19) (Figure 3A). TDI was associated with a significant decrease in maximum early diastolic velocity among CAD patients when compared to those without CAD (MD: -1.91; 95% CI: -2.74 to −1.09; *I*^*2*^ =0%) (Figure 3B). Maximum late diastolic velocity was also significantly decreased among CAD patients when compared to those without CAD (MD: -1.54; 95% CI: -2.33 to −0.75). A random-effects meta-analysis was performed because of significant heterogeneity (*I*^*2*^ =66%), and the mean difference between patients with and without CAD remained statistically significant (MD: -1.57; 95% CI: -2.95 to −0.18) (Figure 3C).

### Sensitivity analyses

Meta-analyses using only those studies that verified both the presence and absence of CAD with angiography showed similar results as the primary analyses. However, there was decreased heterogeneity for the outcome of maximum late diastolic velocity both pre- and post-stress. Additionally, maximum systolic velocity was significantly decreased among CAD patients when compared to those without CAD post-stress, although heterogeneity was still significant (Table 3).

**Table 3:** Summary table with pooled results

**Outcome**	Primary analysis*			Sensitivity analysis of studies using angiography to diagnose or exclude CAD*		
	**N of studies**	**N of patients**	**Pooled Results** – **MD(95% CI); I**^**2**^	**N of studies**	**N of patients**	**Pooled Results** – **MD(95% CI); I**^**2**^
**Pre**-**stress**						
Maximum systolic velocity	5	200	−0.66 (−0.98 to −0.34); 46%	3	132	−0.54 (−0.91 to −0.17); 44%
Maximum early diatolic velocity	6	314	−0.94 (−2.02 to 0.14); 88%	4	246	−0.89 (−2.52 to 0.74); 89%
Maximum late diastolic velocity	6	314	−0.79 (−1.72 to 0.13); 77%	4	246	−0.13 (−0.61 to 0.35); 0%
**Post**-**stress**						
Maximum systolic velocity	3	164	−2.64 (−5.47 to 0.19); 90%	2	133	−3.66 (−7.23 to −0.10); 87%
Maximum early diatolic velocity	3	173	−1.91 (−2.74 to −1.09); 0%	2	142	−1.45 (−2.53 to −0.38); 0%
Maximum late diastolic velocity	3	173	−1.57 (−2.95 to −0.18); 66%	2	142	−2.30 (−3.32 to −1.28); 0%

Abbreviations: *CI* confidence interval, *MD* mean difference.

* Fixed-effects model when I^2^ < 50% and random-effects model when I^2^ ≥ 50%.

### Secondary outcomes

**Figure 4:**
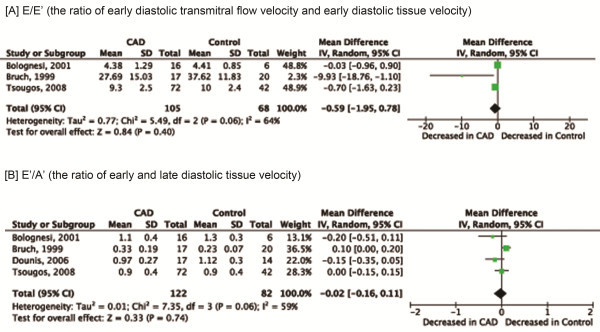
Meta-analysis: Secondary outcomes.

There was not a statistically significant mean difference between E/E' or E'/A' measurements using TDI among CAD patients when compared to those without CAD (Figure 4).

## Discussion

To our knowledge, this is the first systematic review and meta-analysis on the use of TDI for the evaluation of CAD. Our results demonstrated significant differences in TDI measurements in those with and without CAD, including significant decreases in the maximum systolic velocity pre-stress, and maximum early diastolic velocity and maximum late diastolic velocity post-stress.

A correlation between decreased systolic velocity and reduced regional myocardial blood flow has also been demonstrated in animal models [20] and a decreased maximum systolic velocity is highly sensitive and specific for detection of abnormal segmental myocardial motion [21]. In contrast, relatively little data is available specific to maximum early and late diastolic velocities. However, one study demonstrated that reduction of early diastolic velocity was more accurate than maximum systolic velocity for detection of CAD during dobutamine stress echocardiography [22]. A formal evaluation of the test characteristics (sensitivity, specificity, positive and negative predictive value) of TDI was not done because the studies reporting these were done in disparate patient population and used different indices of TDI [23-28].

Our results suggest that decreases in maximal systolic velocity using TDI could be used to detect CAD in resting studies, especially in patients with poor two-dimensional endocardial definition. It could be incorporated into an echocardiogram based CAD diagnostic score, perhaps combined with wall motion score index, to improve the detection of CAD. Wall motion analysis is qualitative, and the addition of TDI adds a quantitative component in the detection of CAD, theoretically leading to improved intra- and inter-reader variability. Although not addressed by these data, abnormal velocities may also signal a more severe degree of CAD than suggested by wall motion analysis alone [29,30].

Measurement of diastolic velocities could also be incorporated into stress testing algorithms, particularly dobutamine stress echocardiography, in which there is time to measure both two-dimensional and TDI parameters. In the ischemic cascade [31], diastolic dysfunction occurs earlier than systolic dysfunction and thus measurement of diastolic velocities by TDI may prove more sensitive than wall motion analysis alone in the detection of CAD during stress testing.

However, the cutoff values for maximum systolic velocity and diastolic velocities which detect CAD are, as yet, unclear. Furthermore, TDI velocity can only be measured in one dimension and is significantly limited by angle dependence, complicating assessment of multiple wall segments (especially more apical segments). The absence of difference in the maximum systolic velocity post-stress with TDI may be a result of this limitation. Speckle tracking is a new technique that measures myocardial motion in every direction, thus overcoming the limitation of angle dependency and reliably quantifying myocardial ischemia in both infarcted and non-infarcted patients [32,33]. TDI velocities are also influenced by tethering and translation, movement of other adjacent structures and blood flow. Despite these limitations, the American Society of Echocardiography/European Association of Echocardiography guidelines acknowledge that TDI has major advantages like ready availability and quantitative evaluation through online measurement of velocities and time intervals [34].

The conclusions of our meta-analysis are limited by the quality and relatively small number of included studies. Another limitation is the significant heterogeneity observed in the meta-analyses of maximum early and late diastolic velocity (pre-stress); and maximum systolic velocity and late diastolic velocity (post-stress). The heterogeneity could be the result of subtle differences in patient populations, segments in which TDI velocities were measured, models of the echocardiography machines used in the assessments, or type of tissue Doppler used (spectral Doppler, which measures the instantaneous velocity, versus color Doppler which measures the modal velocity). The methods of stress testing used (e.g., dipyridamole vs. dobutamine) to diagnose CAD were different in the included studies and present an intrinsic limitation in interpreting the analyses. Sensitivity analyses examining only those studies where CAD was evaluated using angiography showed a reduction in heterogeneity in some analyses (Table 3), suggesting that the method used to diagnosis CAD in the studies may have contributed to the observed heterogeneity. A third limitation is that data on head-to-head comparisons between TDI and other non-invasive imaging modalities or wall motion score analysis for diagnosing CAD are not available. It is thus unclear whether TDI has incremental value for diagnosing CAD when compared with resting (or stress) wall motion abnormalities or EKG abnormalities. However, resting wall motion abnormalities and EKG abnormalities are relatively insensitive for detection of CAD, so TDI may be helpful in improving the sensitivity of these tests. Another limitation is that coronary angiography was not performed in all patients across the studies. However, sensitivity analyses that excluded such studies showed similar meta-estimates. There may also be differences in the proportions of patients with variables like hypertension and left ventricular hypertrophy (LVH) across the studies included in this systematic review, and these differences could account for at least some of the heterogeneity observed across studies in the test characteristics of TDI for diagnosing CAD, as well as differences across studies in E/E' or E'/A' measurements.TDI would be low in patients with LVH or hypertension, potentially leading to false positives in patients with hypertension or LVH who do not have CAD. Thus, if the proportion of these patients differed across studies, then the test characteristics of TDI for diagnosing CAD might differ as a result. The proportion of patients with hypertension ranged from 21% to 58% across the included studies which could account for some of the observed heterogeneity in the test characteristics of TDI for diagnosing CAD. The proportion of patients with LVH was not reported in any study. A final limitation is that none of the above studies examined clinical outcomes, like symptomatic CAD.

In conclusion, our results suggest that TDI has a role in the evaluation of CAD. Future studies should evaluate the incremental value of TDI systolic and diastolic velocities over LV ejection fraction and two dimensional wall motion analysis in the detection of CAD and assessment of its severity.

## Abbreviations

CAD: Coronary artery disease; CI: Confidence interval; LV: Left ventricle/ventricular; MeSH: Medical Subject Heading; MD: Mean difference; TDI: Tissue Doppler imaging.

## Competing interests

None of the authors had any financial or non-financial competing interests

## Authors’ contributions

RA: Conception and design, analysis and interpretation of the data, drafting of the article, final approval of the article. PG: Collection and assembly of data, final approval of the article. JNK: Critical revision of the article for important intellectual content, final approval of the article. TA: Critical revision of the article for important intellectual content, final approval of the article. RD: Critical revision of the article for important intellectual content, final approval of the article. GS: Collection and assembly of data, final approval of the article. CAU: Critical revision of the article for important intellectual content, final approval of the article. All authors read and approved the final manuscript.
